# Alternate Grainy head isoforms regulate *Drosophila* midgut intestinal stem cell differentiation

**DOI:** 10.1038/s41420-025-02496-8

**Published:** 2025-04-29

**Authors:** Nicole Dominado, Rachel Ye, Franca Casagranda, James Heaney, Nicole A. Siddall, Helen E. Abud, Gary R. Hime

**Affiliations:** 1 Department of Anatomy and Physiology, University of Melbourne, Parkville, VIC Australia; 2 Department of Anatomy and Developmental Biology, Biomedicine Discovery Institute, Monash University, Clayton, VIC Australia; 3 Development and Stem Cells Program, Monash Biomedicine Discovery Institute, Monash University, Clayton, VIC Australia

**Keywords:** Intestinal stem cells, Differentiation

## Abstract

Regeneration of the *Drosophila* midgut epithelium depends upon differential expression of transcription factors in intestinal stem cells and their progeny. The *grainy head* locus produces multiple splice forms that result in production of two classes of transcription factor, designated Grh.O and Grh.N. *grainy head* expression is associated with epithelial tissue and has roles in epidermal development and regeneration but had not been examined for a function in the midgut epithelium. Here we show that null mutant clones had a limited effect on intestinal stem cell (ISC) maintenance and proliferation but surprisingly specific loss of all Grh.O isoforms results in loss of ISCs from the epithelium. This was confirmed by generation of a new Grh.O class mutant to control for genetic background effects. Grh.O mutant ISCs were not lost due to cell death but were forced to differentiate. Ectopic expression of a Grh.N isoform also resulted in ISC differentiation similar to loss of Grh.O function. Grh.O expression must be tightly regulated as high level ectopic expression of a member of this isoform class in enteroblasts, but not ISCs, resulted in cells with confused identity and promoted excess proliferation in the epithelium. Thus, midgut regeneration is not only dependent upon signalling pathways that regulate transcription factor expression, but also upon regulated mRNA splicing of these genes.

## Introduction

To provide barrier function between organs and their surrounds epithelial tissues must be able to repair or replace damaged cells [1]. The *Drosophila* midgut epithelium is an excellent model to study the molecular processes that regulate maintenance of epithelial stem cells and their differentiation. The differentiated cells of the midgut epithelium are constantly replaced by a population of regenerating ISCs scattered along the basal surface of the epithelium. ISC divisions produce daughter ISC and transient enteroblast (EB) progenitors. EBs can differentiate into either an absorptive enterocyte (EC) or a secretory enteroendocrine cell (EE) [2–4] although data indicate that ECs and EEs arise from different progenitors and EE cells can directly originate from ISCs primed to form EE cells [5–8]. ECs are large polyploid cells that make up the bulk of the midgut epithelia, interspersed with hormone-producing EE cells, which regulate peristalsis [9], cell fate [5, 7] and intestinal stem cell (ISC) proliferation [10, 11].

The Grainy head (Grh) family of transcriptional regulators is conserved across metazoan lineages and functions in epidermal barrier formation, wound healing, tubulogenesis and cancer. Appearance of the *grainy head* gene family coincides with the evolution of epithelia, highlighting the importance of this family for epithelial regulation [12].

*Drosophila* has a solitary *grh* gene whereas mammals have evolved three *Grhl* genes, *Grhl-1, Grhl-2*, and *Grhl-3*. Studies in both *Drosophila* and mice have demonstrated that specific Grh proteins are essential for formation and maintenance of epithelial tissues. The vertebrate studies have produced complex results with different members of the Grh family associated with induction of differentiation and in some cancer studies, stemness [13–17].

**Fig. 1:**
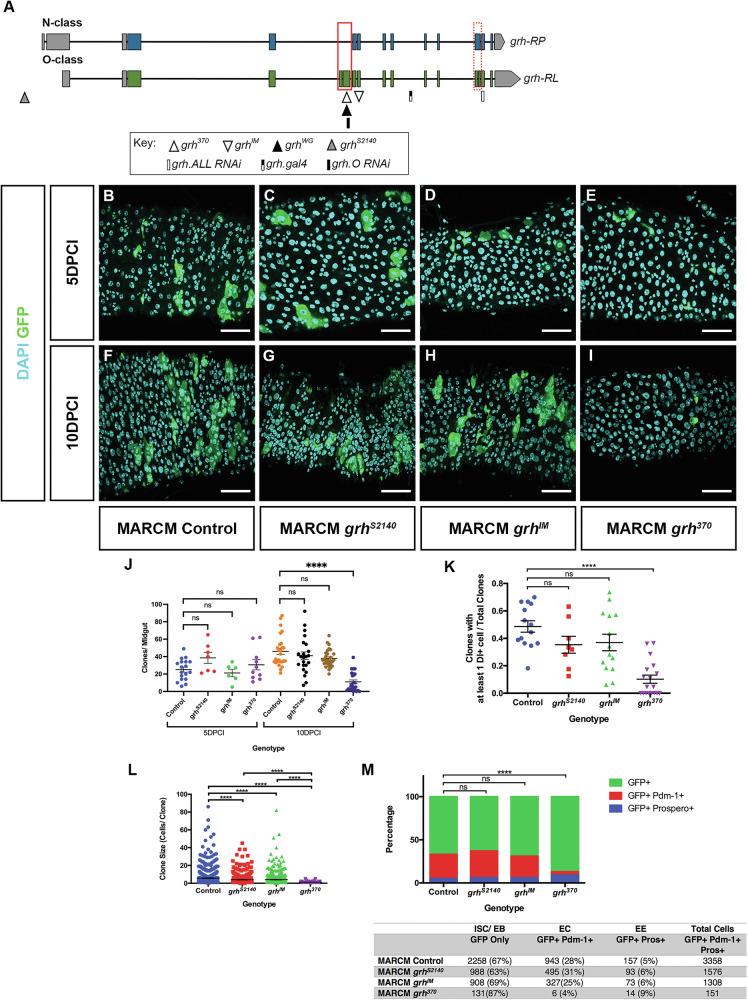
*grh* O-isoforms maintain ISC identity. **A** Schematic of *grh* mRNA transcripts. The single *grh* gene is alternatively spliced to produce two classes of transcripts based on the splicing of exons 4 and 5 (red box). Transcripts with exons 4 and 5 are classed as O-isoforms while those without these exons are classed as N-isoforms. Transcripts denoted with an apostrophe lack 30 amino acids in exon 12 (red dotted box). *grh.RP* (*grh.N)* and *grh.RL (grh.O’)* are shown as representatives of the two classes. Positions of mutations are indicated by triangles and the grh.Gal4 insertion by a black and white bar. Sequences that were utilized for RNAi constructs are indicated by bars. **B**–**I** Representative images of GFP (green) marked control, *grh*^*S2140*^*, grh*^*IM*^ *and grh*^*370*^ MARCM clones at 5PDCI and 10DPCI. Scale Bar 40 µm. **J** Quantification of the mean number of clones per midgut at 5 and 10 days post clone induction (DPCI). MARCM control (5DPCI: *n* = 18 midguts, 10DPCI *n* = 26 midguts) and *grh* null (*grh*^*S2140*^ *–* 5DPCI: *n* = 7 midguts, 10DPCI n = 23 midguts; *grh*^*IM*^ – 5DPCI: *n* = 6 midguts 10DPCI: *n* = 28 midguts) O-specific mutant *grh*^*370*^ (5DPCI: *n* = 10 midguts, 10DPCI: *n* = 25 midguts) clones are lost at 10DPCI. (Mean ± SEM, One-way ANOVA with Dunnett’s test, ns= not significant, ****p < 0.0001). **K** Proportion of MARCM clones at 10DPCI containing at least a single Dl+ ISC in control (*n* = 14), *grh*^*S2140*^ (*n* = 8), *grh*^*IM*^ (*n* = 14) and *grh*^*370*^ (*n* = 18) midguts. In comparison to control clones, there is a significant decrease in the fraction of *grh*^*370*^ clones containing ISCs. (Mean ± SEM, One-Way ANOVA with Tukey’s test, ns= not significant, ****p < 0.0001). **L** *grh*^*370*^ MARCM clones affect ISC proliferation. Control clones are larger in size compared to *grh* mutant clones at 10DPCI. Clonal size was calculated by counting the number of cells within each clone in control and *grh* mutants. Control – *n* = 770 clones, 26 midguts. *grh*^*S2140*^ *–* *n* = 526 clones, 15 midguts. *grh*^*IM*^*-* *n* = 554 clones, 21 midguts. *grh*^*370*^ – *n* = 269 clones, 25 midguts. (Mean ± SEM, One-Way ANOVA with Tukey’s Test, ***p = 0.0002, ****p < 0.0001). **M** *grh*^*370*^ MARCM clones affect ISC differentiation. Quantification of the cellular composition of MARCM clones immunostained with Prospero and Pdm-1. The number of ECs (GFP+ Pdm-1+), EE cells (GFP+ Prospero+) and ISC/EBs (GFP+) for control and *grh* mutant clones was quantified for each genotype and percentage values calculated. (*χ*^2^ Test, control vs. *grh*^*S2140*^ *p* = 0.8325, control vs *grh*^*IM*^ *p* = 0.8650 and control vs. *grh*^*370*^ ****p < 0.0001).

In *Drosophila*, the single *grh* (also known as *Elf-1*/*NTF-1)* gene is alternatively spliced. *grh* transcripts can be classed into two groups with those containing exons 4 and 5 known as O-isoforms (Grh.O and Grh.O’), whereas transcripts without these exons are classed as N-isoforms (Grh.N and Grh.N′) [18] (Fig. 1A). These isoforms have now been renamed in Flybase as Grh.RJ = Grh.O, Grh.RL = Grh. O′, Grh.RP = Grh.N and Grh.RH = grh.N’. O-isoforms have been reported as being restricted to neural tissues in third instar larvae with N-isoforms associated with epithelial maintenance and wound repair in non-neural tissues [18–21]. Analysis of Grh in the midgut would facilitate an understanding of how this gene family may function in regenerative epithelia. Here we show that both isoforms are expressed at very low levels in the midgut epithelium where they play different roles in regulating ISC maintenance, proliferation and differentiation.

## Results

### Grainyhead O-isoforms maintain ISCs by preventing differentiation

We examined two *grh* amorphic (null) alleles, *grh*^*S2140*^ and *grh*^*IM*^, in addition to a hypomorphic allele, *grh*^*370*^ which only disrupts collective O-isoform function (both Grh.RJ and Grh.RL’) [18] and leaves all N-isoforms intact. Homozygotes of each allele exhibit lethaliity prior to adulthood, hence the MARCM system [22] was utilized to generate GFP-marked homozygous *grh* mutant clones in posterior midgut tissue.

Initial analysis of clones 5–10 days post clonal induction (DPCI) revealed that the number of *grh*^*370*^ clones per midgut was less than that of control and *grh* null clones (Fig. 1B–J) at 10DPCI. Given that clones arise and are maintained by ISCs, a loss of GFP marked clones over time would suggest that Grh O class-isoforms are required for the maintenance of ISCs. To further investigate this hypothesis, control and *grh* clones 10DPCI were immunostained with the ISC marker, Delta (Dl). In comparison to controls (0.49 ± 0.04 clones), there was a slight but non-significant decrease in the proportion of clones with at least 1 Dl+ cell in *grh* null mutants (*grh*^*S2140*^*:* 0.35 ± 0.06; *grh*^*IM*^*:* 0.37 ± 0.06 clones) (Fig. 1K and Supplementary Fig. 1). In contrast, a reduction was observed in the O-isoform specific *grh*^*370*^ with only 0.10 ± 0.03 clones containing at least one ISC.

It would be expected that as *grh*^*370*^ clones at 10DPCI have fewer ISCs they would exhibit a decrease in proliferation relative to controls, which can be measured by counting the number of cells within a clone [22]. Thus, using clone size as a measure of ISC proliferation confirmed the size of *grh*^*370*^ clones was significantly smaller, averaging 1 cell per clone compared to controls, with a mean of approximately 6 cells per clone (Fig. 1L). *grh* null clone sizes were also reduced relative to the control (Fig. 1L), but not to the extent of the *grh*^*370*^ clones and null cones did not show a reduction in ISC number (Fig. 1K) indicating an unusual genetic property of the *grh*^*370*^ allele.

To distinguish the cell types that make up multicellular *grh* clones, the EC marker Pdm-1 and the EE marker Prospero (Pros) were assayed to determine the percentages of cells within clones with an EC (Pdm-1+GFP+), EE (Pros+ GFP+) and ISC/EB fate (GFP+ only). The cellular composition between control and *grh* null clones (Fig. 1M) was similar (ECs: 25–31%, EEs: 5–6% and ISC/EBs: 63–69%) suggesting that complete loss of Grh does not affect differentiation. The specific loss of O-isoforms also did not prevent differentiation as *grh*^*370*^ cells positive for Pdm-1 (4% of *grh*^*370*^ cells) and Pros (9% of *grh*^*370*^ cells) could be detected, albeit Pdm-1 was detected at a much lower percentage than in control clones. Consequently, an increase in ISC/EBs (87% of *grh*^*370*^ cells) was observed. As loss of Grh O class-isoforms results in a loss of ISCs, it is likely that a majority of these GFP+ only cells are EBs. These data imply that loss of O-isoform function results in differentiation of ISCs to EBs but also a reduced rate of EB to EC differentiation.

**Fig. 2:**
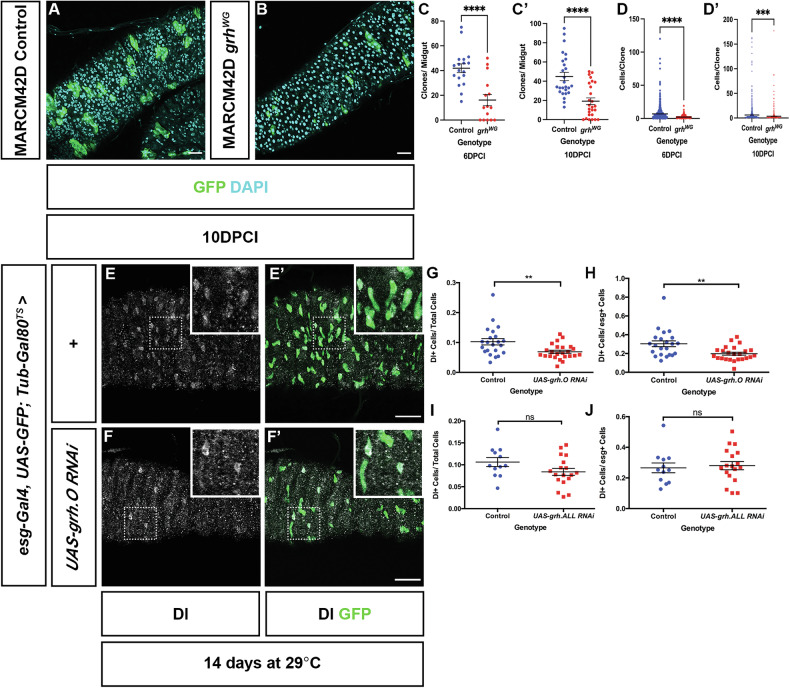
*grh*^*WG*^ MARCM clones and O-class isoform-specific RNAi also result in loss of progenitor cells. Confocal images of control (**A**) and *grh*^*WG*^ (**B**) MARCM clones maintained at 25 C. Scale Bar 40μm. **C**, **C**′ Quantification of the number of clones per midgut in control (6DPCI: *n* = 18 midguts 10DPCI: *n* = 26 midguts) and *grh*^*WG*^ (6DPCI: *n* = 15 midguts, 10DPCI: *n* = 26 midguts) MARCM clones at 6DPCI and 10DPCI. A reduction in the number of clones in *grh*^*WG*^ midguts is observed at both time points quantified. (Mean ± SEM, Unpaired Student's *T*-test, ****p < 0.0001). **D**, **D**′ Quantification of clonal size between control and *grh*^*WG*^ MARCM clones. At both 6DPCI and 10DPCI, *grh*^*WG*^ clonal size, measured by counting the number of cells per clone is smaller than that of control clones. Control – 6DPCI: *n* = 754 clones, 18 midguts; 10DPCI: *n* = 1169 clones, 26 midguts. *grh*^*WG*^ – 6DPCI: *n* = 242 clones, 15 midguts; 10DPCI: *n* = 499 clones, 26 midguts. (Mean ± SEM, Unpaired Student's *T*-test, ***p = 0.0003, ****p < 0.0001). **E**, **F**′ Confocal images of control and *esg*^*TS*^ > *UAS-grh.O-class RNAi* midguts. ISCs are marked by Dl and progenitor cells marked with GFP. Scale Bar 40 µm. Inset images are magnified views of regions enclosed by dotted boxes. **G** Quantification of Dl+ ISC cells in control (*n* = 22 midguts) and *esg*^*TS*^ > *UAS-grh.O-class RNAi* (*n* = 24 midguts) midguts showed a decrease in the proportion of Dl+ cells over total cell number. (Mean ± SEM, Unpaired Student's *T*-test with Welsh’s Correction, **p = 0.0078). **H** Proportion of esg+ cells that are Dl+ ISCs in control (*n* = 22 midguts) and *esg*^*TS*^ > *UAS-grh.O-class RNAi* (*n* = 24 midguts). (Mean ± SEM, Unpaired Student's *T*-test with Welsh’s Correction, **p = 0.0049). **I** Quantification of the proportion of Dl+ ISCs over total cell number in control (*n* = 12 midguts) and *esg*^*TS*^ > *UAS-grh.ALL RNAi* (*n* = 18 midguts) show no significant difference between the two genotypes. (Mean ± SEM, Unpaired Student's *T*-test with Welsh’s Correction, ns not significant). **J** The proportion of esg+ cells that are Dl+ ISCs remain at similar levels in control (*n* = 12 midguts) and *esg*^*TS*^ > *UAS-grh.ALL RNAi* (*n* = 18 midguts) midguts. (Mean ± SEM, Unpaired Student's *T*-test with Welsh’s Correction, ns = not significant).

The more severe phenotype observed in *grh*^*370*^ MARCM clones suggested that either loss of O-class isoforms has a more severe effect than complete loss of Grh function, or that a second site mutation on the *grh*^*370*^ chromosome was responsible for the loss of ISCs. We therefore sought to validate the observed phenotype in two ways. Firstly, we generated another *grh* mutation *(grh*^*WG*^*)* that specifically mutated all O-isoforms by incorporation of 3-frame stop codons within exon 5 (see Materials and Methods and Supplementary Fig. 2). Similar to the decrease observed in the number of *grh*^*370*^ clones, the number of *grh*^*WG*^ clones per midgut also decreased at 6DPCI (16.13 ± 4.55 clones) and 10DPCI (19.19 ± 3.44 clones) in comparison to controls (41.89 ± 3.54 and 44.96 ± 4.34, at 6DPCI and 10DPCI, respectively) (Fig. 2A–C). The number of cells per clone also significantly decreased in *grh*^*WG*^ clones compared to controls (Fig. 2D).

As the phenotype recapitulated that observed with *grh*^*370*^, the loss of *grh*^*WG*^ in MARCM clones confirms that loss of Grh O class-isoforms are responsible for the loss of ISCs and not a secondary unknown mutation in the *grh*^*370*^ background.

The requirement for Grh O-isoforms in maintaining ISCs was further confirmed via RNA interference. We generated a short hairpin RNA targeting exon 5 and therefore specifically all O-class isoforms (see Materials and Methods). Efficiency of knockdown is shown in Supplementary Fig. 3. This was expressed in ISCs and EBs using the *esg-Gal4, UAS-GFP; Tub-Gal80* (henceforth known as *esg*^*TS*^) driver. After 14 days at the permissive temperature quantification of Dl+ cells in control midguts showed an average proportion of 0.10 Dl+ cells of the total cells. This average proportion significantly decreased to 0.07 in *esg*^*TS*^ > *UAS-grh.O RNAi*. Further analyses quantifying the ratio of ISCs to EBs (by examination of the proportion of Esg+ cells that were also Dl+) show a decrease of ISCs in *esg*^*TS*^ > *UAS grh.O RNAi* midguts containing a mean proportion of 0.20 Dl+ cells compared to an average of 0.30 Dl+ in controls (Fig. 2E–H). This suggests that the majority of the relative proportion of GFP-marked ISC/EBs (hereafter termed Esg+) in *esg*^*TS*^ > *UAS grh.O RNAi* midguts are EBs and that knock down of Grh O-isoforms results in premature differentiation of ISCs to EBs. No difference was observed using RNAi that targets all isoforms (Fig. 2I, J), further indicating that it is the specific loss of Grh.O that results in loss of ISCs. We also used the esgF/O system to drive expression of *UAS-grh.O RNAi* in ISCs/EBS and again observed a reduction in Dl+ cells, indicating loss of ISCs (Supplementary Fig. 4).

### Ectopic expression of N isoforms results in differentiation

**Fig. 3:**
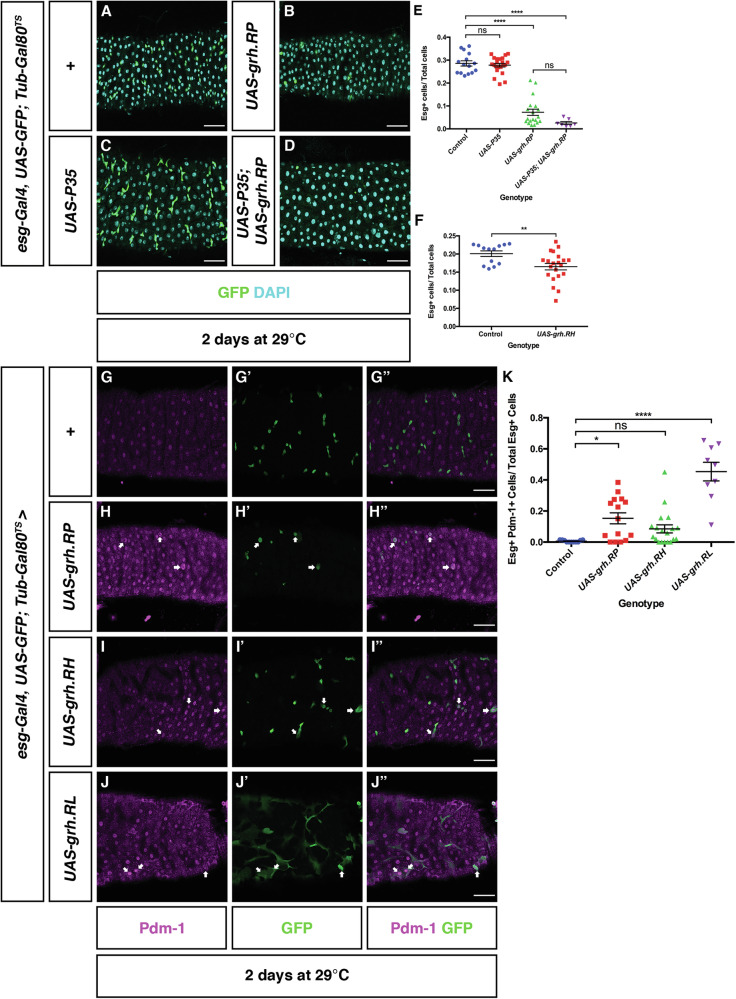
Ectopic expression of the *grh.RP* (N-class isoform) promotes differentiation. Representative images of control midguts (**A**), midguts overexpressing Grh.RP (**B**), the apoptotic inhibitor P35 (**C**) and midguts co-expressing P35 and Grh.RP (**D**) in progenitor cells. Scale Bar 40 μm. **E** Quantification of the number of progenitor cells in control (*n* = 15 midguts) midguts, midguts over expressing Grh.RP (*n* = 19 midguts), P35 (*n* = 22 midguts) and midguts co-expressing Grh.RP and P35 (*n* = 7 midguts) shows that expression of p35 was not able to rescue loss of progenitor cells. (Mean ± SEM, One-Way ANOVA with Tukey’s Test, ns not significant, ****p < 0.0001). **F** Quantification of the proportion of ISC/EBs in control and *esg*^*TS*^ > *UAS-grh.RH (N*′ *isoform)* midguts. The proportion of progenitors in *esg*^*TS*^ > *UAS-Grh.RH* midguts (*n* = 21) have decreased in comparison to control midguts (*n* = 13). (Mean ± SEM, Unpaired Student's *T*-test, **p = 0.0088). Confocal images of control (**G**) and midguts ectopically expressing Grh.RP (**H**) and Grh.RH (**I**) and Grh.RL (Grh.O-class) (**J**) in ISC/EBs. Compared to controls, GFP+ progenitor cells expressing Grh.RP, Grh.RH or Grh.RL have increased cell size and also express the EC marker, Pdm-1 (arrows). Scale Bar 40 μm. **K** The proportion of esg+ progenitor cells expressing Pdm-1 in midguts increases in midguts over expressing N-class isoforms Grh.RP (*n* = 15), Grh.RH (*n* = 9) and O-class isoform Grh.RL (*n* = 8) in comparison to control midguts (*n* = 11). While the proportion of esg+ progenitor cells expressing Pdm-1 slightly increased in midguts overexpressing Grh.RH (*n* = 19), it was not statistically significant compared to controls. (Mean ± SEM, one-way ANOVA with Tukey’s Test, ns not significant, *p = 0.0107, ****p < 0.0001).

The more severe phenotype observed in O-class isoform-specific mutants, *grh*^*370*^ *and grh*^*WG*^, could be explained if the N- and O- class isoforms had differing roles in the regulation of ISCs. The structure of the *grh* gene did not permit us to generate a loss of N-class alleles as N-isoform exons are shared with O-isoforms. Hence we decided to imbalance the N/O ratio by increasing N-isoform activity. grh.RP (grh.N-class) was overexpressed in ISC/EBs using *esg*^*TS*^. After two days at the non-permissive temperature, there was a significant reduction in the relative proportion of Esg+ (or progenitor cells) in *esg*^*TS*^ > *UAS-*grh.RP (grh.N-class) midguts compared to controls (Fig. 3A,B & E). To test that the decrease in progenitors was not due to sequences outside of exons 4–5 we showed that ectopic expression of another N-class isoform, grh.RH (which contains a variant 3′ exon) also resulted in a loss of Esg+ cells (Fig. 3F). Thus, ectopic expression of Grh N-isoforms in ISC/EBs results in the loss of ISC/EBs.

To investigate if the reduction of ISC/EBs due to the overexpression of grh.RP (grh.N-class) is a result of cell death, control and *esg*^*TS*^ > *UAS*− Grh.RP midguts were immunostained with an antibody generated against human activated Caspase 3, which also detects apoptotic cells in *Drosophila* [23, 24]. An activated Caspase 3 signal was not detected in control and *esg*^*TS*^ > *UAS-*Grh.RP midguts suggesting that apoptosis is not the cause of ISC/EB loss (Supplementary Fig. 5A–C). This was further confirmed by co-expressing the apoptotic inhibitor P35 [25] with *UAS-*Grh.RP (grh.N-class) using the esg^TS^ driver (Fig. 3C–E). Co-expression of P35 with Grh.RP for two days at the permissive temperature was not sufficient in preventing the loss of Esg+ cells with the total proportion remaining at a similar level to the expression of Grh.RP alone. The ISC/EB proportion in midguts only expressing P35 was comparable to controls demonstrating that expression of P35 does not affect ISC/EB number. Together these data illustrate that loss of ISC/EBs due to Grh.N-class overexpression is not a consequence of apoptosis.

Instead of apoptosis, the loss of progenitors could be a result of differentiation. Indeed, the proportion of Dl+ cells in *esg*^*TS*^ > *UAS*-Grh.RP midguts showed a decreasing trend in comparison to controls (0.01 vs. 0.08, respectively) (Supplementary Fig. 6A,B, D). Moreover, some of the few Esg+ cells present that ectopically express Grh.RP (grh.N-class) have large nuclei characteristic of ECs suggesting that they have differentiated (Fig. 3H). In comparison to control midguts (Fig. 3G), there was an increase in the proportion of Esg+ cells expressing the EC marker, Pdm-1, in midguts ectopically expressing Grh.RP in ISC/EBs (Fig. 3K). While this trend was also observed when Grh.RH (also grh.N-class but with an alternatively spliced 3′ exon) was ectopically expressed using *esg*^*TS*^, quantification showed that it was not statistically significant. These data indicate that increased expression of N-class isoforms in ISC and EBs induces the process of differentiation down the EC lineage.

### *Grainy head* is expressed in the adult midgut

**Fig. 4:**
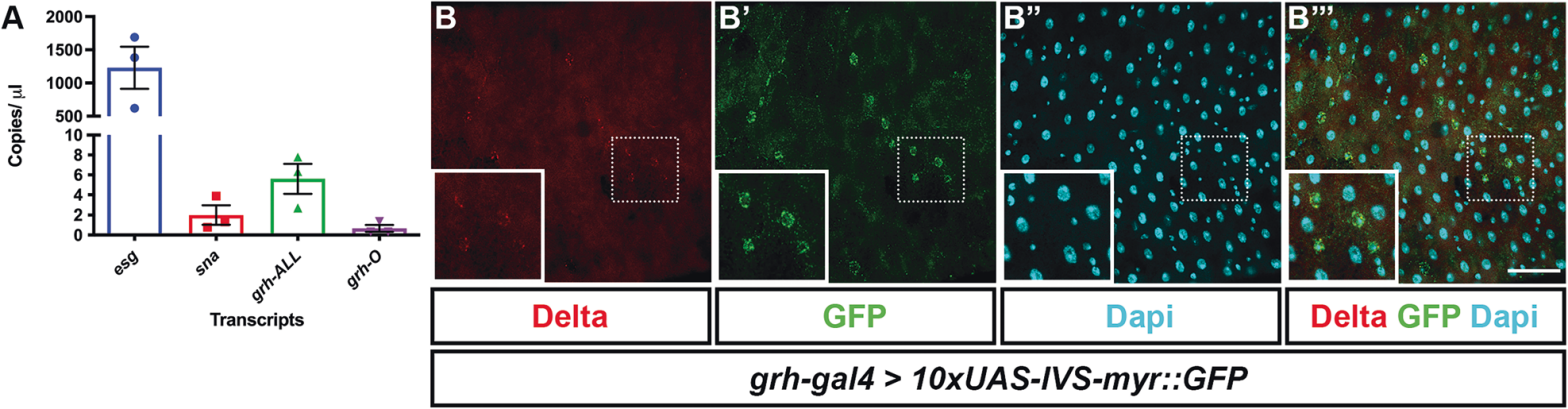
*grh* is expressed in the midgut. **A** Quantification of *esg*, s*na* and *grh* mRNA transcripts in 5–7-day old adult *w*^*1118*^ midguts shows that in comparison to known ISC/EB marker *esg*, *grh* transcripts are expressed at much lower levels but is more comparable to that of ISC regulator, *sna*. mRNA quantification was conducted using biological triplicates (*n* = 15 midguts for each replicate) with each replicate represented by individual data points. Bar graph represents mean ± SEM of data points. **B** Midguts of *grh-gal4* > *10xUAS-IVS-myr::GFP*. A GFP signal was observed in Delta-positive ISCs. Boxes show magnified regions demarcated by dotted box. Scale Bar 40μm.

To determine if *grh* is expressed in the adult midgut, we designed primers to target subsets of *grh* mRNA transcripts (Supplementary Table 1). Additionally, primers for *esg*, an ISC/EB marker [2] and *sna*, a gene known to function in ISC and EBs [26] were used as positive controls. As expected, ddPCR (which allows absolute quantification of transcript levels) conducted on *w*^*1118*^ control midguts showed robust *esg* expression (Fig. 4A) (1231 copies/μl ±318: Mean ± SEM) with expression of *sna* barely detectable at a much lower level (1.99 copies/μl ±0.96). Primers detecting all *grh* transcripts showed a slightly higher expression level of 5.60 copies/μl ±1.51. Transcripts responsible for the formation of O-isoform class expression were also detected at a much lower level of 0.86 copies/μl ± 0.58.

To investigate the spatial resolution of Grh, immunofluorescent staining of phenotypically wild-type midguts utilizing several Grh-specific antibodies was performed (Supplementary Table 3). However, a signal could not be detected using four of the five antibodies [27–31] although we could detect signal if we ectopically expressed Grh in the midgut (Supplementary Fig. 7A–H′). While an immunofluorescent signal was detected using the final Grh antibody tested, this was later shown to be non-specific, with the signal remaining in GFP-marked *grh* null mutant clones (Supplementary Fig. 7E-F). The antibodies may have been unable to detect low level protein expression, and the mutant analysis clearly indicates a role for Grh in the midgut, so we obtained a grh-GFP strain [32] which also showed no expression in the posterior midgut (Supplementary Fig. 8A–A″) although it expressed strongly in imaginal discs (Supplementary Fig. 8B). We reasoned that damage to the midgut could potentially induce *grh* expression so examined grh-GFP at 2, 4, 24 and 48 h after treatment with dextran sodium sulfate (DSS) but again could not observe expression of GFP (Supplementary Fig. 8C–J′). We then obtained a strain that contains a transposable element carrying the Gal4 open reading frame inserted into a Grh intron flanked by exons 9 and 10 of the Grh.RJ transcript. This strain, *grh*^*1249-G4*^, expresses Gal4 under the influence of adjacent enhancer sequences [33]. We crossed *grh*^*1249-G4*^ to 10xUAS-IVS-myr::GFP and could detect GFP expression in cells that co-labelled with Delta (Fig. 4B-B‴). These data suggest that Grh transcripts are present in the midgut and that Grh transcription is active in ISCs.

### Ectopic Grh O-class isoforms promote a delay in differentiation that results in cells having characteristics of EBs and ECs

**Fig. 5:**
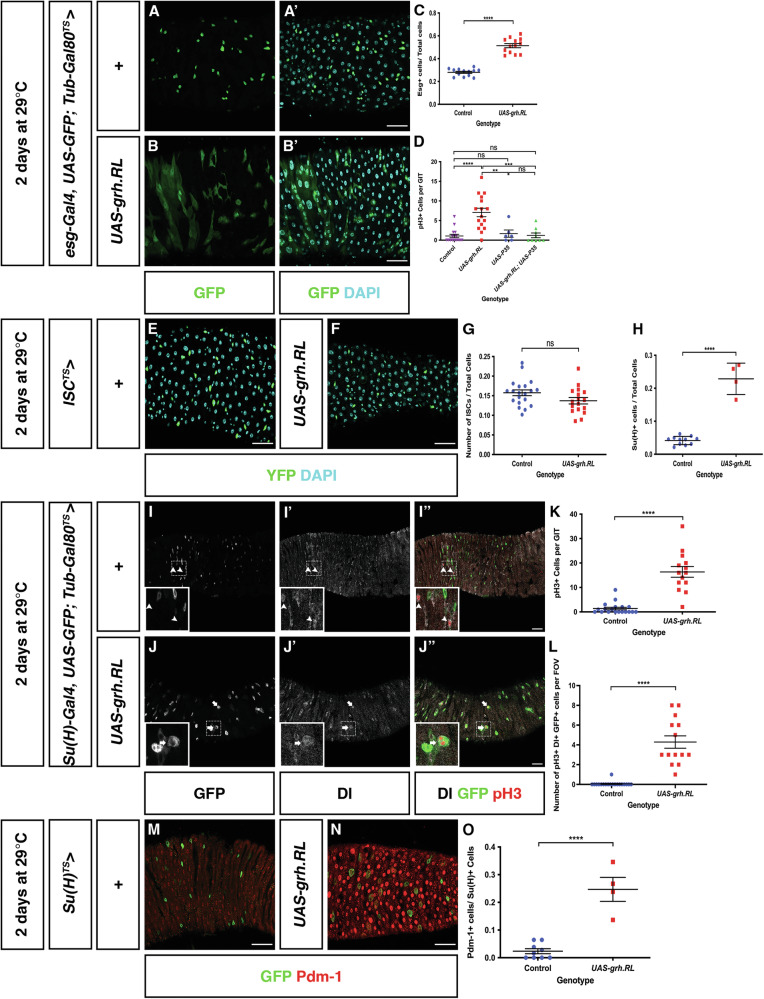
Over expression of an O-class isoform results in both ISC maintenance and differentiation. Representative images of control midguts (**A**) and midguts overexpressing Grh.RL in progenitor cells (**B**). Scale Bar 40 μm. **C** In comparison to control midguts (*n* = 13) there is an increase in the proportion of esg+ cells in midguts over expressing Grh.RL (*n* = 13). (Mean ± SEM, Unpaired Student's *T*-test with Welsh’s Correction, ****p < 0.0001). **D** An increase in the mitotic index as measured by the number of pH3+ cells are observed in midguts ectopically expressing Grh.RL (*n* = 16). This increase in mitotic index is rescued by the co-expression of the apoptotic inhibitor p35 with Grh.RL (*n* = 9) with its mitotic index returning to levels seen in control midguts (*n* = 16) and midguts only expressing p35 (*n* = 6). (Mean ± SEM, One-Way ANOVA with Tukey’s Test, **p = 0.0027, ***p = 0.0001, ****p < 0.0001). **E**- **F** Confocal images of control midguts and midguts ectopically expressing Grh.RL in only ISCs (green). Ectopically expressing Grh.RL in ISCs does not appear induce differentiation with the YFP+ ISCs remaining at a similar size to YFP+ cells in control midguts. Scale Bar 40 μm. **G** The proportion of ISCs in control midguts (*n* = 20) and midguts ectopically expressing Grh.RL (*n* = 17) remain at similar level. (Mean ± SEM, unpaired Student's *T*-test with Welsh’s Correction, *p* = 0.0749). **H** In comparison to controls (*n* = 11 midguts), an increase in GFP+ EBs (express Su(H) EB marker) is observed in midguts ectopically expressing Grh.RL (*n* = 4). (Mean ± SEM, Unpaired Student's *T*-test with Welsh’s Correction, ****p < 0.0001). **I**, **J** Representative images of control midguts (**I**) and midguts overexpressing Grh.RL (**J**) in EBs (driven from Su(H)Gal4). In control midguts, ISCs are solely marked by Dl with mitotically active ISCs marked by pH3 (arrowheads) whereas overexpression of Grh.RL in EBs induces EB expression of Dl and EB mitotic division (arrows). Scale Bar 40μm. Dotted boxes outline magnified regions. **K** Comparison of the mitotic index in control midguts (*n* = 19) and midguts overexpressing Grh.RL (*n* = 14) in EBs. An increase in the mitotic index is observed in midguts overexpressing Grh.RL in EBs. (Mean ± SEM, Unpaired Student's *T*-test, ****p < 0.0001). **L** Quantification of the number of EBs undergoing mitotic division. (Mean ± SEM, Unpaired Student's *T*-test, ****p < 0.0001). **M**, **N** Confocal images of control midguts and midguts overexpressing Grh.RL in EBs immunostained with EC marker, Pdm-1. Whereas GFP+ EBs in controls rarely express Pdm-1, an increase in *Su(H)*^*TS*^ > UAS-Grh.RL cells are positive for Pdm-1 is observed. Scale Bar 40 μm. **O** The proportion of EBs expressing Pdm-1 increases in *Su(H)*^*TS*^ > UAS-Grh.RL midguts (*n* = 4) in comparison to control midguts (*n* = 11). (Mean ± SEM, Unpaired Student's *T*-test, ****p < 0.0001).

We have demonstrated that loss of Grh.O isoforms results in loss of ISCs, and ectopic expression of Grh.N-class isoforms promotes ISC differention but we were unsure if this latter effect was simply a result of high level expression of any isoform class. To examine the effect of specifically elevating O-class isoform levels, we ectopically expressed Grh.RL in ISC/EBs using the *esg*^*TS*^ driver. Increased expression of Grh.RL resulted in an increase in the proportion of Esg+ cells when compared to controls (Fig. 5A–C) and hence demonstrated a phenotype that differed from ectopic expression of N-class isoforms. This phenotype could represent an increase in either ISCs or EBs. To differentiate the two cell types, control and *esg*^*TS*^ > *UAS-* Grh.RL midguts were immunostained with the ISC marker, Delta (Dl). Analysis of *esg*^*TS*^ > *UAS-*Grh.RL revealed that the proportion of Esg+ cells stained with Dl was elevated compared to controls suggesting an increase in ISCs (Supplementary Fig. 6A–D). Interestingly, cells labelled with both Dl and GFP in *esg*^*TS*^ > *UAS*-Grh.RL midguts appeared enlarged compared to controls (Supplementary Fig. 6E), a property of ECs which undergo endoreplication and an increase in cellular size. Therefore, the EC marker, Pdm-1, was examined in control and *esg*^*TS*^ > UAS-Grh.RL midguts. While Esg+ cells that express Pdm-1 were not observed in controls, they were commonly observed in *esg*^*TS*^ > *UAS-*Grh.RL (Fig. 3J-K). The expression of Pdm-1 in *esg*^*TS*^ > *UAS-*Grh.RL cells indicates that increased Grh.RL activity also accelerates differentiation (similar to ectopic expression of N-class isoforms), although the maintenance of Dl expression shows the cells retain some ISC properties. Many of these cells also have an elongated morphology that suggests they have some properties associated with EBs, indicating an intermediate status with features of ISCs, EBs and ECs (Fig. 3J–J″).

The accumulation of progenitor cells with confused identity is reminiscent to the age-related decline in epithelial structure that leads to cell death [34]. Typically, the induction of apoptosis in the midgut leads to compensatory ISC proliferation [35]. This was indeed observed in midguts overexpressing Grh.RL (Grh.O-class) using either *Esg*^*TS*^ or *Su(H)*^*TS*^ (Fig. 5D, K) with the mitotic index, measured by counting the number of Phospho-histone 3 positive cells, increasing to a mean of 7 compared to a control mean of 1 (Fig. 5D). Moreover, the increase in mitotic index was rescued (returning to a mean of ~1) when apoptotic inhibitor P35 was co-expressed with Grh.RL. Ectopic expression of Grh.RL also resulted in caspase activation (Supplementary Fig. 5). These data suggest that abnormal accumulation / ectopic expression of Grh.RL in progenitor cells results in cells with confused identity and ultimately cell death. Consequently, it is EB/EC cell death that stimulates ISC proliferation.

Our data suggest that overexpression of Grh.RL can induce both markers of differentiation and ISC identity. In order to determine the effect of Grh.RL overexpression in ISCs alone, we utilized *esg-Gal4, UAS-YFP; Su(H)-Gal80, Tub-Gal80*^*TS*^ (hereafter termed *ISC*^*TS*^) which will inhibit Gal4-mediated expression in EBs. Ectopic expression of Grh.RL in ISCs did not result in an increase in ISC number with the number of ISCs remaining at an equivalent level to that of controls (Fig. 5G). Moreover, the increase in Grh.RL expression in ISCs did not enhance premature differentiation into ECs as GFP+ nuclear size remained at a similar size to that of controls (Fig. 5E, F) and was very different to the phenotype observed when Grh.RL was expressed in ISCs/EBs (compare to Fig. 5B-B′).

The above data suggest that the retention of ISC-like properties after ectopic expression of Grh.RL using *esg-Gal4*^*TS*^ may be entirely due to expression within EBs so we specifically investigated the function of Grh.RL ectopic expression in EBs by using *Su(H)GBE-Gal4, UAS-GFP; Tub-Gal80*^*TS*^ (hereafter termed *Su(H)*^*TS*^). Ectopic expression of Grh.RL in EBs led to an increase in the proportion of GFP+ cells in comparison to controls (Fig. 5H). The increase in GFP+ (presumed EB) number appears to be as a result of an increase in the mitotic index of *Su(H)*^*TS*^ > *UAS-*Grh.RL midguts (Fig. 5I–K). Somewhat surprisingly, a number of these mitotically active cells were GFP+ (and Dl+) suggesting that ectopic expression of Grh.RL in EBs may facilitate EB cell division (Fig. 5J, L). Given that cell division is characteristic of ISCs, it is possible that these cells may have some ISC-like identity.

In addition to maintaining ISC-like identity in *esg*^*TS*^*>* Grh.RL midguts, the overexpression of Grh.RL was also able to accelerate differentiation into the EC lineage. This ability was also investigated in EBs. Analysis of *Su(H)*^*TS*^ *>* Grh.RL midguts showed that there was an increase in the number of EBs expressing the EC marker, Pdm-1 (Fig. 5M–O) suggesting that high-level ectopic expression of Grh.RL, similar to the N-class isoforms, is also able to accelerate expression of an EC marker, however, it results in cells with confused identity as some share markers of ECs and EBs (GFP) and some cells are in a mitotic state and express the ISC-marker, Delta.

## Discussion

Here we have provided evidence that Grh is required to maintain ISCs in the *Drosophila* midgut. The effects of loss and gain of Grh function on epithelial development are summarized in Supplemetary Fig. 9. Surprisingly, it is the function of the O-class isoforms that is crucial for ISC maintenance. Generation of *grh*^*370*^ midgut clones, including a new CRISPR-generated allele, demonstrated that ISCs that do not express O-class isoforms are lost from the epithelium. It is unusual for a hypomorphic mutation to exhibit a more severe phenotype than a null but this is not unprecedented in genes that express multiple isoforms. For example, the murine *Trp73* gene encodes two major classes of isoforms, resulting from alternative initiation sites, termed TAp73 and ΔNp73 [36]. Each isoform has unique properties and it appears to be the balance of isoforms in a cell that determines phenotypic outcome. Specific isoform knockouts in mice exhibit unique phenotypes. *TAp73*^*−/−*^ mice develop tumours not observed in *Trp73*^*−/−*^ nulls [37].

*grh*^*370*^ clones lose stem cells but increase the proportion of GFP+ Pdm-1- Pros- cells compared with control clones suggesting that they have an increased proportion of EBs, or that ISCs differentiate rather than being lost via apoptosis (Supplementary Fig. 9A, B). These data imply that Grh O-isoforms maintain ISCs by preventing them from prematurely differentiating. Interestingly, over-expression of Grh.O (Grh.RL) in only ISCs did not induce an increase in ISC number suggesting that it alone cannot specify ISC identity. This consistent with previous work identifying Grh as a pioneer factor that does not directly activate gene expression but opens chromatin at sites of epithelial enhancers to allow access to other transcriptional regulators [38].

The mechanism of why loss of O-class isoforms (*grh*^*370*^ and *grh*^*WG*^ alleles) results in a more severe phenotype with respect to ISC maintenance than null alleles is yet to be determined. The organization of Grh isoforms (they share exons except for the O-class specific exons 4 and 5, and another alternative splicing event near the C-terminus that is found in both O-class and N-class isoforms) means that it is not possible to generate specific N-class loss of function alleles. Overexpression of N-class isoforms (Grh.RP or Grh.RH) resulted in a loss of ISCs and EBs via forced differentiation (Supplementary Fig. 9C). However, differentiation proceeded in *grh* null clones. This suggests that N-isoforms, while they may facilitate differentiation if ectopically expressed, are not critical in the absence of O-isoforms and other factors may compensate for their loss. High-level ectopic expression of a Grh.N-class isoform may force ISC differentiation by allowing access of differentiation factors to target genes that are normally repressed in ISCs.

Overexpression of the O-class isoform, Grh.RL, in ISCs/EBs (driven from *Esg-Gal4*^*TS*^) resulted in cells with confused identities (Supplementary Fig. 9D). While a majority of the Esg+ cells expressed the ISC marker, Dl, they also exhibited increased cell size and expressed the EC marker, Pdm-1. This could indicate dual roles for O-isoforms in both ISC maintenance and EC differentiation but the phenotype may also be a result of the high level of ectopic expression produced by GAL4. What is clear is that Grh.RL (O-class) can induce ISC marker expression in EBs and this phenotype is qualitatively different from overexpressing Grh N-class proteins.

The mechanism of how Grh isoforms exert their specificity is yet to be determined. The O-specific sequences do not appear to be conserved in vertebrate GRHL proteins despite them exhibiting differential splicing [39], however differential activities of the vertebrate proteins may offer the suggestion that isoform-specific functions observed in *Drosophila* have evolved into separate gene functions in vertebrates. Further work will determine how GRH/GRHL proteins and isoforms co-operate in regulating epithelial stem cell maintenance and differentiation.

It is curious as to why *grh* is expressed at such low levels in the midgut epithelium. We were only able to detect expression within ISCs. A very tight control of *grh* expression may be needed to maintain ISCs but also permit EB differentiation. It is possible that a network of transcriptional regulators act to facilitate midgut differentiation that may be at, or beyond, the resolution of detection by current RNAseq pipelines. This underscores the importance of genetic screens that may be capable of detecting phenotypes associated with genes that could be potentially ignored from transcriptome analyses.

## Materials and methods

### *Drosophila* stocks and husbandry

Fly stocks were maintained on a standard culture medium at 25 °C unless otherwise specified. Mated female flies were exclusively analyzed throughout this study, due to differences in male and female ISC behaviour [40]. A detailed list of fly strains used in this study are listed in Supplementary Table 2. Flies containing the *Tub-Gal80*^*TS*^ allele were crossed and maintained at the non-permissive temperature of 18 °C. Following eclosion, 3–5-day old flies were transferred to the permissive temperature of 29 °C for further analysis.

### Generation of marked clones

The Mosaic Analysis with Repressible Cell Marker System (MARCM) was used to generate positively marked GFP homozygous clones. Unless otherwise stated, MARCM crosses were established and maintained at 18 °C. 3–5 day old adult flies of the genotypes *UAS-cd8 GFP, hs-FLP/ +; frt42DtubGal80/ frt42D; tub-Gal4/+, UAS-cd8 GFP, hs-FLP/ +; frt42DtubGal80/ frt42Dgrh*^*S2140*^*; tub-Gal4/+, UAS-cd8 GFP, hs-FLP/+; frt42DtubGal80/ frt42Dgrh*^*IM*^*; tub-Gal4/+, UAS-cd8 GFP, hs-FLP/+; frt42DtubGal80/ frt42Dgrh*^*370*^*; tub-Gal4/+* were heat shocked for 1 h in a 37 °C running water bath. Flies were then returned to 18 °C to minimize the incidence of leaky MARCM clones. Intestines were analyzed 5–10 days after clonal induction.

### Generation of *UAS-grh.O-class* RNAi Line

Short hairpin design was conducted using *grh* exon 5 sequence with top candidate selected using DSIR website [41]. Selected hairpin, 5′-CGGGATCAGACAAATATCCAA-3′ was cloned into pWALIUM20 [42] with potential plasmids verified by Sanger sequencing. Verified plasmid was then injected into embryos (BestGene Inc. Co) and a stable transgenic line made.

### Generation of *grh*^*WG*^ allele

CRISPR mediated mutagenesis, performed by WellGenetics Inc. was used to insert 3-frame stop codons in *grh* exon 5 to generate a C-terminal truncation only affecting O-isoforms (*grh-RJ, RL, RN and RO)*. Briefly, DNA plasmids containing hs-Cas9, *grh* gRNAs targeting exon 5 and a cassette containing 2 loxP sites, selection marker 3xP3-RFP, two homology arms and 3-frame stop codons were injected into *w*^*1118*^ embryos. F1 flies carrying 3xP3-RFP selection marker were then validated by PCR, sequencing and backcrossing to *grh*^*IM*^ and *Df(grh)*.

### Immunostaining

Adult mated female *Drosophila* midguts were dissected in Phosphate Buffered Saline (PBSx1). Dissected midguts were then fixed in 4% formaldehyde for 1 h or overnight at 4 °C. Following fixation, midguts were then washed 3× in PBT (PBS + 0.1% Triton-X100) for 5 min each and blocked for 1 hour in PBTH (PBS + 0.1% Triton X100 + 5% Horse Serum). Midguts were then incubated in the following primary antibodies: chicken anti-GFP (1:2000, AbCam, ab307275), mouse anti-Delta (1:100, DSHB, C594.9B), rabbit anti-Pdm-1 (1:2000, Xiaohang Yang), rabbit anti-pH3 (1:5000, Upstate, now Merck, #06-570), mouse anti-β-Galactosidase (1:20, DSHB), chicken anti-β-Galactosidase (1:2000, AbCam, ab134435), rabbit anti-β-Galactosidase (1:2000, Cappel, now MP Biomedical, #55976), mouse anti-GRH (1:5, Sarah Bray), rat anti-GRH (1:500, Stefan Thor), rabbit anti-GRH (1:200, William McGinnis /1:1000, Melissa Harrison), rabbit anti-Activated Caspase 3 (D175) (1: 200, Cell Signaling Technology,) overnight at 4 °C. After primary antibody incubation, the samples were washed 3× for 5 min in PBT and incubated in corresponding Alexa Fluor (1:500, Invitrogen) secondary antibodies for 2 h. This was then followed by a DAPI wash for 20 min and 3× washes in PBT for 5 minutes each. Samples were then mounted in 80% glycerol. All steps were carried out at room temperature unless otherwise stated.

### Image acquisition and analysis

Images were acquired on the Zeiss LSM800 or LSM880 Confocal Microscopes as serial optical sections (z-stacks) of 1024 ×1024 resolution. Only the first 500 μm adjacent to the pyloric ring was imaged and analyzed throughout this study. This was to ensure consistent comparison of regions between midgut samples and the prevention of confounding results due to regional midgut differences [43, 44].

Clonal cell counts were conducted using Bitplane Imaris software to render 3D reconstructions of intestines. Imaris Spot and Surface functions were then utilized to quantify and map cell nuclei to clones.

Cell counts were performed using FIJI/ImageJ software. FIJI was also used to process images and generate maximal intensity z-projections displayed in figures. Adobe Photoshop used to compile figure panels. Statistical analysis and graphs were created using Graphpad PRISM.

### Droplet digital PCR

Droplet Digital PCR (ddPCR) was used to for expression analysis during this study. Taqman gene expression assays were purchased from Thermofischer Scientific (Supplementary Table 1). RNA was extracted either from at least 15 midguts per genotype using the Qiagen RNeasy Mini Kit (Cat no. 74104). RNA quality was analyzed on the Agilent Tapestation 2200. RNA with an RNA Integrity Number (RIN) above 8 was used for analysis. RNA quantification was carried out on a Qubit 4 Fluorometer. 500 ng of RNA was then used for cDNA synthesis using the Bioline Sensifast cDNA Synthesis Kit (Cat no. BIO-65053) as per the manufacturer’s instructions.

### Dextran sodium sulfate (DSS) damage

To induce injury, flies were starved for 2 h at 29 °C. Following starvation, flies were transferred to an empty vial containing a cotton ball soaked in 5% sucrose with or without 3% DSS (MP Biomedicals, cat no. 160110) for the required treatment times.

## Supplementary information

Supplementary files

## Data Availability

All data generated or analyzed during this study are included in this published article and in its supplementary information file.
