# Supplementary material for: Alternate Grainy head isoforms regulate Drosophila midgut intestinal stem cell differentiation
Source: Cell Death Discov. 2025 Apr 29;11:206. doi: 10.1038/s41420-025-02496-8 (PMC12037896; doi:10.1038/s41420-025-02496-8)
Supplement: Supplementary file 1 — Supplementary files [file 41420_2025_2496_MOESM1_ESM.pdf]

Supplementary tables, figures, figure legends and references for Dominado et. al.

Tables (Supplementary):

Supplementary Table 1: TaqMan Gene Expression Assays

**Gene Name**	**Assay ID**
sna	Dm01841564_s1
esg	Dm01841264_s1
grh-ALL	Dm01816396_m1
grh-RJ, RL (O-class isoforms)	Dm01841950_m1
RpL32	Dm02151827_g1
RpL11	Dm01842483_g1

Supplementary Table 2: Fly Strains Used in this Study

**Fly Strain**	**Source**
w^{1118}	Bloomington Drosophila Stock Centre BDSC_3605
grh^{S2140}*/CyO*	BDSC_10460
Su(H)GBE-Gal4, UAS-GFP / CyO	Stephen Hou
Delta-LacZ	BDSC_11651
grh^{06850}*/CyO*	BDSC_12325
esg^{TS} *(esgGal4, UAS GFP; TubGal80*^{TS}*/ [SM6B-TM6B])*	Kieran Harvey
MARCM42D (hs-FLP, UAS-CD8GFP; FRT42DtubGal80/ CyO; Tub-Gal4/TM6B)	Leonie Quinn
esgGal4, TubGal80^{TS}*, UAS-GFP; UAS-FLP, Act>CD2>Gal4/ TM6B*	Jerome Korzelius
b pr cn grh^{370}*bw/CyO*	Alex Gould
cn^1 grh^{IM} bw^1*/ SM6a*	BDSC_3270

UAS-grh.RL (O' isoform)	BDSC_42227/42228
UAS-grh.RP (N isoform)	Christos Samakovlis
esg;Su(H)::Gal80 (w; esg-Gal4, UAS-2xYFP/ CyO; Su(H)Gal80, TubGal80^{TS}/ TM3)	Leanne Jones
UAS-grh.RH (N' isoform)	This Study
UAS-10x-UAS-IVS-myr-GFP	BDSC_32197
UAS-grh.O-class RNAi	This Study
UAS-grh.ALL RNAi	Vienna Drosophila Resource Center v101428
UAS-P35	BDSC_5073
grh^{WG}/ CyO	This Study
grh$^{1249\text{-}G4}$ (grh-Gal4)	BDSC_65637

Supplementary Table 3: Grainyhead antibodies used in this study.

Name	Clonality	Target Region	Reference
GRH Antibody 1	Polyclonal	N-terminus	(Nevil *et al.*, 2017)
GRH Antibody 2	Polyclonal	C-terminus	(Harrison *et al*, 2011)
GRH Antibody 3	Polyclonal	C-terminus	(Kim & McGinnis, 2011)
GRH Antibody 4	Monoclonal	Unknown	(Bray *et al.*, 1989)
GRH Antibody 5	Polyclonal	C-terminus	(Baumgardt *et al.*, 2009)

Supplementary Figure Legends

Supplementary Figure 1. A reduction in ISC numbers was observed in *grh^{370}* MARCM clones.

A-D''') Representative confocal images of control **(A-A''')**, *grh^{S2140}* **(B-B''')**, *grh^{IM}* **(C-C''')** and *grh^{370}* **(D-D''')** MARCM clones immunostained with GFP (clone marker) and Delta (ISC marker). Insets depict magnified regions outlined by dashed boxes. GFP positive clones are outlined

(dotted line) within insets and arrowheads mark Delta positive cells inside outlined clones. Scale Bar 40μm.

Supplementary Figure 2. Validation of *grh*^{WG} allele

A) PCR Gel showing incorporation of knock in STOP-RFP cassette in the correct orientation from three heterozygous transgenics 18296B, 18296C and 18296D. Expected band sizes 1368bp (Upstream PCR) and 1387bp (Downstream PCR) was observed in all samples.

B) BLAST sequence similarity report comparing 18296B Upstream PCR sequence with STOP-RFP donor plasmid. Sequences of 3-frame stop codons (purple shading) and loxP (blue shading) was observed in sample 18296B and was correctly inserted in the *grh* coding exon. Allele 18296B was used in this paper and renamed *grh*^{WG}.

Supplementary Figure 3. Analysis of UAS-*grh* RNAi efficiency.

A) Quantification of *grh*.O-class mRNA transcripts in control and *tub-Gal4*^{TS}> *UAS-grh.O RNAi* third instar larval heads. A reduction of *grh*-O mRNA transcripts was observed. (Mean ± SEM, Unpaired T-test with Welch's Correction, ***p=0.0001).

B) Primers that could detect all *grh* mRNA transcripts were used to quantify *grh* transcripts in control and *tub-Gal4*^{TS}> *UAS-grh.ALL RNAi* third instar larval heads. A reduction in *grh* mRNA transcripts was detected. (Mean ± SEM, Unpaired T-test with Welch's Correction, *p=0.0144).

Supplementary Figure 4. Continuous knockdown of Grh.O-class isoforms leads to a reduction of ISC numbers.

A-B''') Representative images of control **(A)** and *esgF/O > UAS-Grh.O RNAi* **(B)** midguts immunostained with the ISC marker, Delta, nuclei marker, DAPI and GFP to mark progenitor cells. Insets depict magnified areas marked by dashed boxes. Scale Bar 40μm.

C) Quantification of progenitor cells in control (n=16) and *esgF/O > UAS-Grh.O RNAi* (n=13) midguts did not show a significant difference in the proportion of progenitor cells between the two genotypes. (Mean ± SEM, Unpaired students T-test, ns = not significant).

D) A reduction in the proportion of Dl+ ISCs over total cell number was observed in *esgF/O > UAS-Grh.O RNAi* when compared to control midguts. (Mean ± SEM, Unpaired students T-test, *p=0.0145).

E) A reduction in the proportion of Dl+ ISCs over esg+ progenitor cells was also observed in *esgF/O > UAS-Grh.O RNAi* in comparison to control midguts. (Mean ± SEM, Unpaired students T-test, *p=0.0387).

Supplementary Figure 5. Over expression of Grh.N does not cause cell death

A-C) Confocal images of control midguts **(A-A''')** midguts over expressing Grh-RP (N class) **(B-B''')** and Grh-RL (O-class) **(C-C''')** in ISC/EBs. Activated caspase 3 was detected in *esg*^{TS}> *UAS*-Grh.RL midguts but not in control and *esg*^{TS}> *UAS*-Grh.RP midguts. Scale Bar 40μm.

D-D''') Representative image of midgut expressing cell death gene, *reaper* in ECs and immunostained with activated caspase 3 as a positive control. Scale Bar 40μm.

Supplementary Figure 6. Ectopic expression of Grh.RL (Grh.O class) in progenitor cells results in cells with confused identity.

A-C'') Confocal images of control midguts **(A-A'')**, *esg*^{TS}> *UAS*-Grh.RP (Grh.N-class) midguts **(B-B'')** and *esg*^{TS}> *UAS*-Grh.RL (Grh.O-class) midguts **(C-C'')** immunostained with Delta (ISC marker), DAPI (nuclei marker) and GFP (ISC/EB marker). Scale Bar 40μm.

D) Quantification of Dl+ ISCs over total cells shows an increase in the proportion of ISCs in *esg*^{TS}> *UAS*-Grh.RL midguts (n=9) but not in *esg*^{TS}> *UAS*-Grh.RP midguts (n=12) when compared to control midguts (n=8). (Mean ± SEM, One-Way ANOVA with Dunnett's Test, *p=0.0276, ns= not significant).

E) Comparison of the average Dl+ GFP+ cell nuclei size between control midguts (n=8), *esg*^{TS}> *UAS-Grh.RP* midguts (n=8) and *esg*^{TS}> *UAS-Grh.RL* midguts (n=9) showed increased nuclei size only in *esg*^{TS}> *UAS-Grh.RL* midguts. (Mean ± SEM, One-Way ANOVA with Dunnett's Test, **p=0.0012, ns= not significant).

Supplementary Figure 7. Grh protein expression could not be discerned using antibodies

A-D') Adult midguts immunostained with four different Grh antibodies. Grh staining was not observed in phenotypically wild type midguts. ISCs were labelled by the reporter *Dl-LacZ* while *Su(H)GBE-Gal4>UAS-GFP* was used to mark EBs. Scale Bar 40μm.

E-F') Adult *esg*^{TS}> *UAS*-Grh.RH midguts immunostained with the same four Grh antibodies used in (A-D). Ectopic expression of Grh.RH resulted in a moderate loss of Esg+ cells but retained enough progenitor cells for observation of Grh immunostaining. All four antibodies labelled ISC/EB cells. Scale Bar 40μm.

I-J') A fifth Grh antibody detected a cytoplasmic Grh (red) signal in the midgut that co-localized with Prospero positive EE cells (magenta). However, this signal is not specific to Grh in the midgut as it can be detected in both control and homozygous *grh* null mutant MARCM clones (green). Scale Bar 40μm.

Supplementary Figure 8. Grh immunostaining was not detectable in *grh-GFP* midguts.

A-A") Confocal image of *grh-GFP* midguts immunostained with GFP antibody. A GFP signal was not detected. Scale Bar 40μm.

B) Representative image of a *grh-GFP* third instar larval central nervous system immunostained with a GFP antibody. A GFP signal was observed in the eye disc (dotted outline), the brain and the ventral nerve cord. Scale Bar 40μm.

C-J') Confocal images of control and DSS damaged *grh-GFP* midguts immunostained with GFP. A GFP signal was not observed in 2 hours **(C-D')**, 4 hours **(E-F')**, 24 hours **(G-H')** and 48 hour **(I-J')** treated midguts. Scale Bar 40μm.

Supplementary Figure 9. Summary of Grh isoform activities in the midgut.

A) Wild type levels of Grh isoforms results in normal ISC maintenance and EB to EC differentiation B) Loss of Grh.O isoforms (Grh.O isoform KO) result in loss of ISCs and increased numbers of EBs suggesting that O-isoforms are required to maintain ISCs. C) Ectopic overexpression of Grh N isoforms (Grh.RP/RH isoform OE) result in forced differentiation to ECs and loss of ISCs/EBs. D) In contrast, overexpression of an O-isoform (Grh.RL isoform OE) leads to accumulation of cells with characteristics of ISCs (Delta expression, mitotic activity), EBs (Su(H)Gal4 activity) and ECs (enlarged size, Pdm-1 expression) suggesting that they have a confused identity.

Supplementary References

Baumgardt M, Karlsson D, Terriente J, Diaz-Benjumea FJ, Thor S (2009) Neuronal subtype specification within a lineage by opposing temporal feed-forward loops. *Cell* 139: 969-982

Bray SJ, Burke B, Brown NH, Hirsh J (1989) Embryonic expression pattern of a family of Drosophila proteins that interact with a central nervous system regulatory element. *Genes Dev* 3: 1130-1145

Harrison MM, Li XY, Kaplan T, Botchan MR, Eisen MB (2011) Zelda binding in the early Drosophila melanogaster embryo marks regions subsequently activated at the maternal-to-zygotic transition. *PLoS Genet* 7: e1002266

Kim M, McGinnis W (2011) Phosphorylation of Grainy head by ERK is essential for wound-dependent regeneration but not for development of an epidermal barrier. *Proc Natl Acad Sci U S A* 108: 650-655

Nevil M, Bondra ER, Schulz KN, Kaplan T, Harrison MM (2017) Stable Binding of the Conserved Transcription Factor Grainy Head to its Target Genes Throughout Drosophila melanogaster Development. *Genetics* 205: 605-620

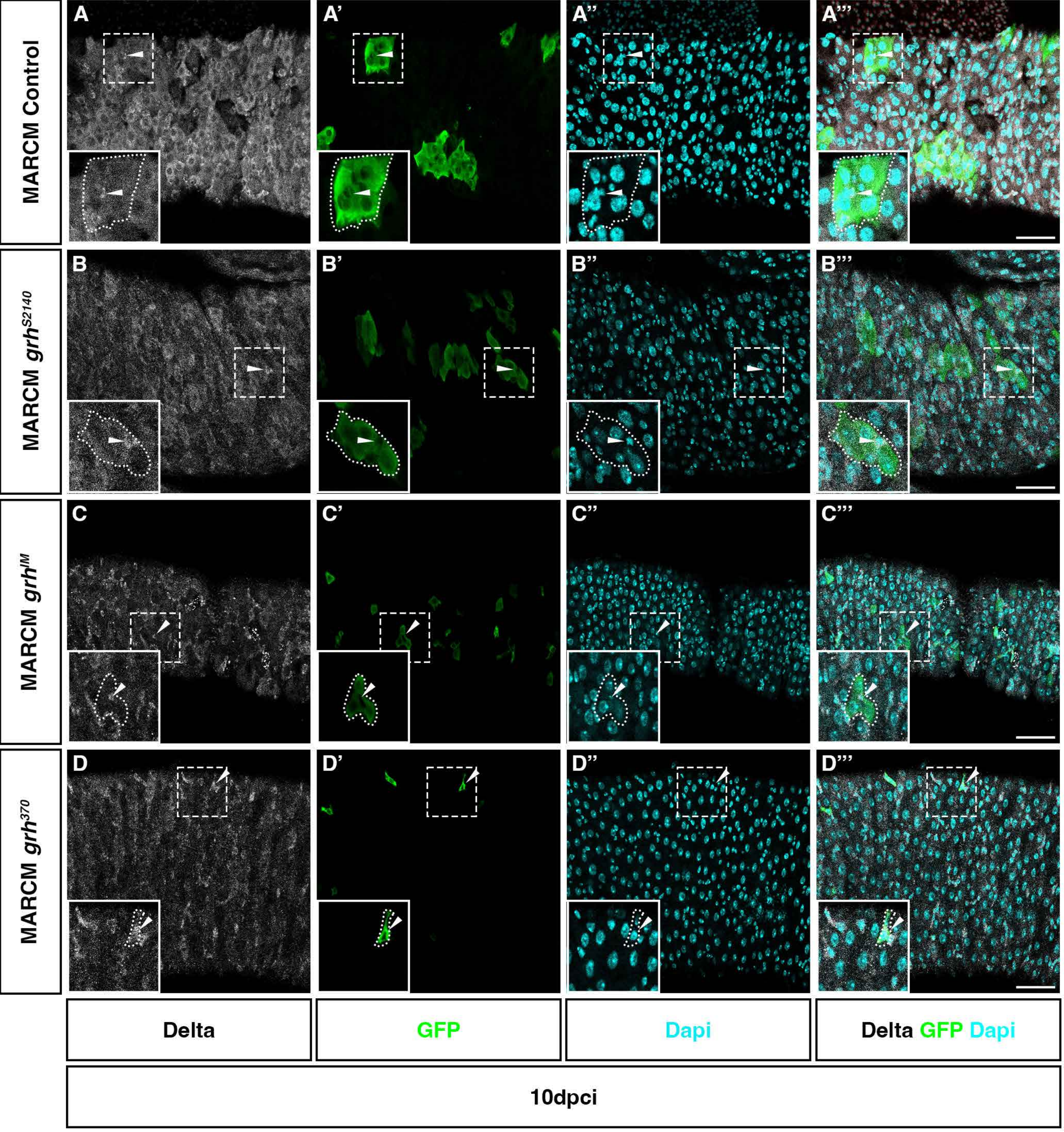

Supplementary figure 1

A

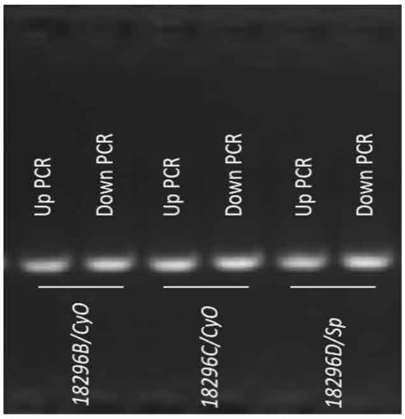

B

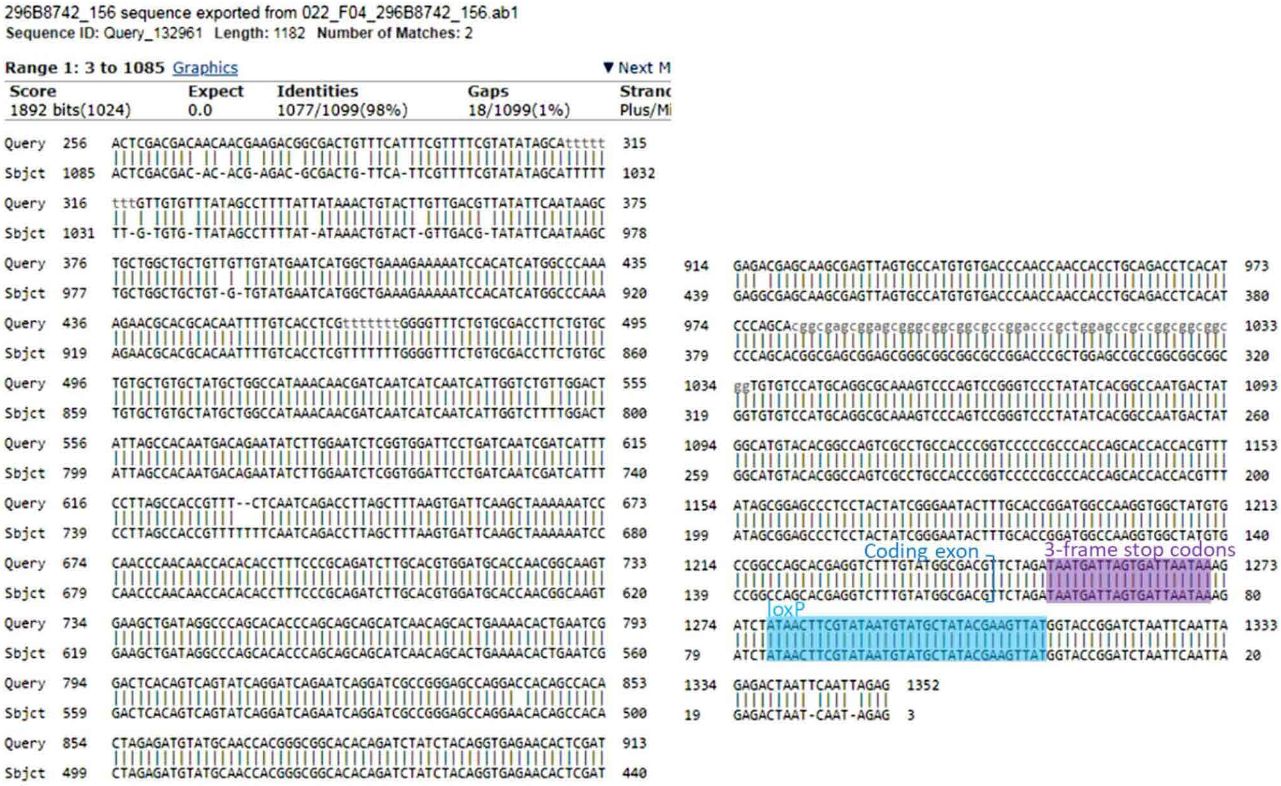

Supplementary figure 2

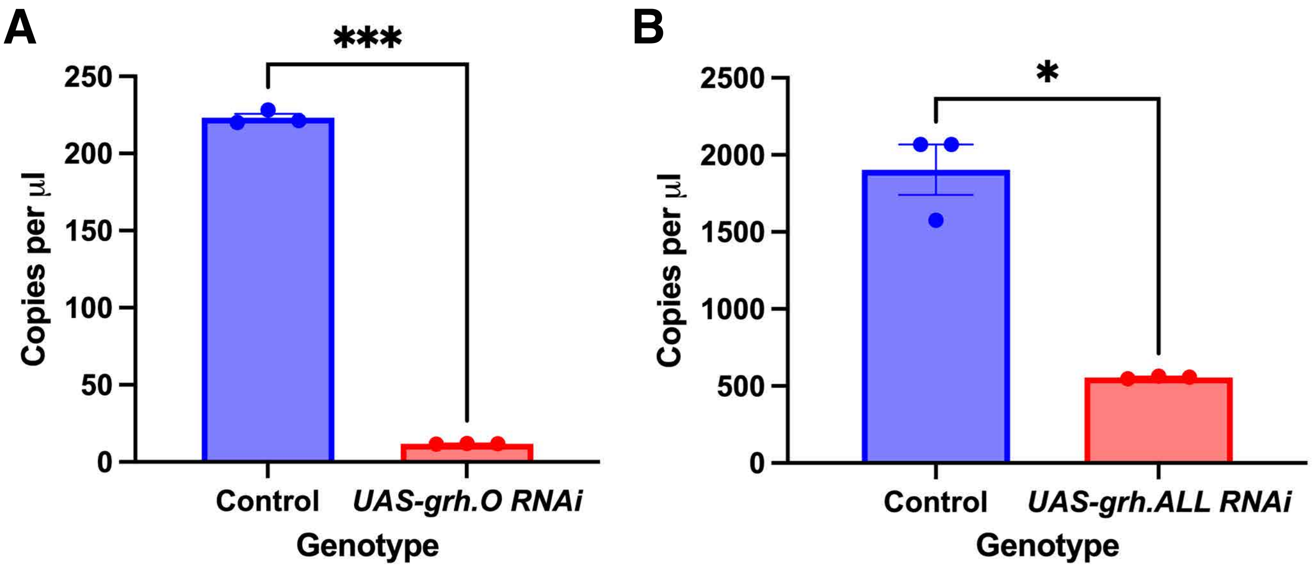

Supplementary figure 3

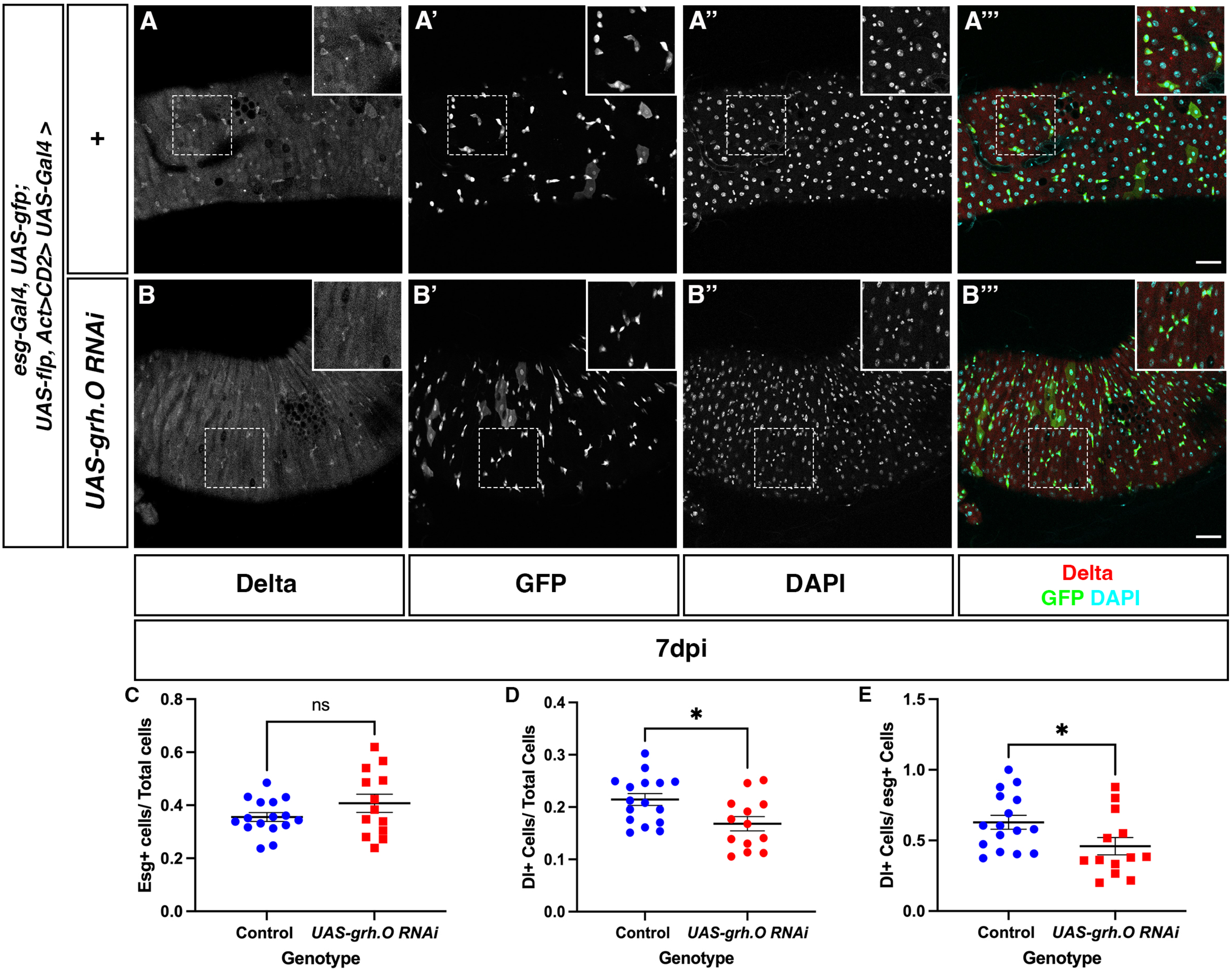

Supplementry figure 4

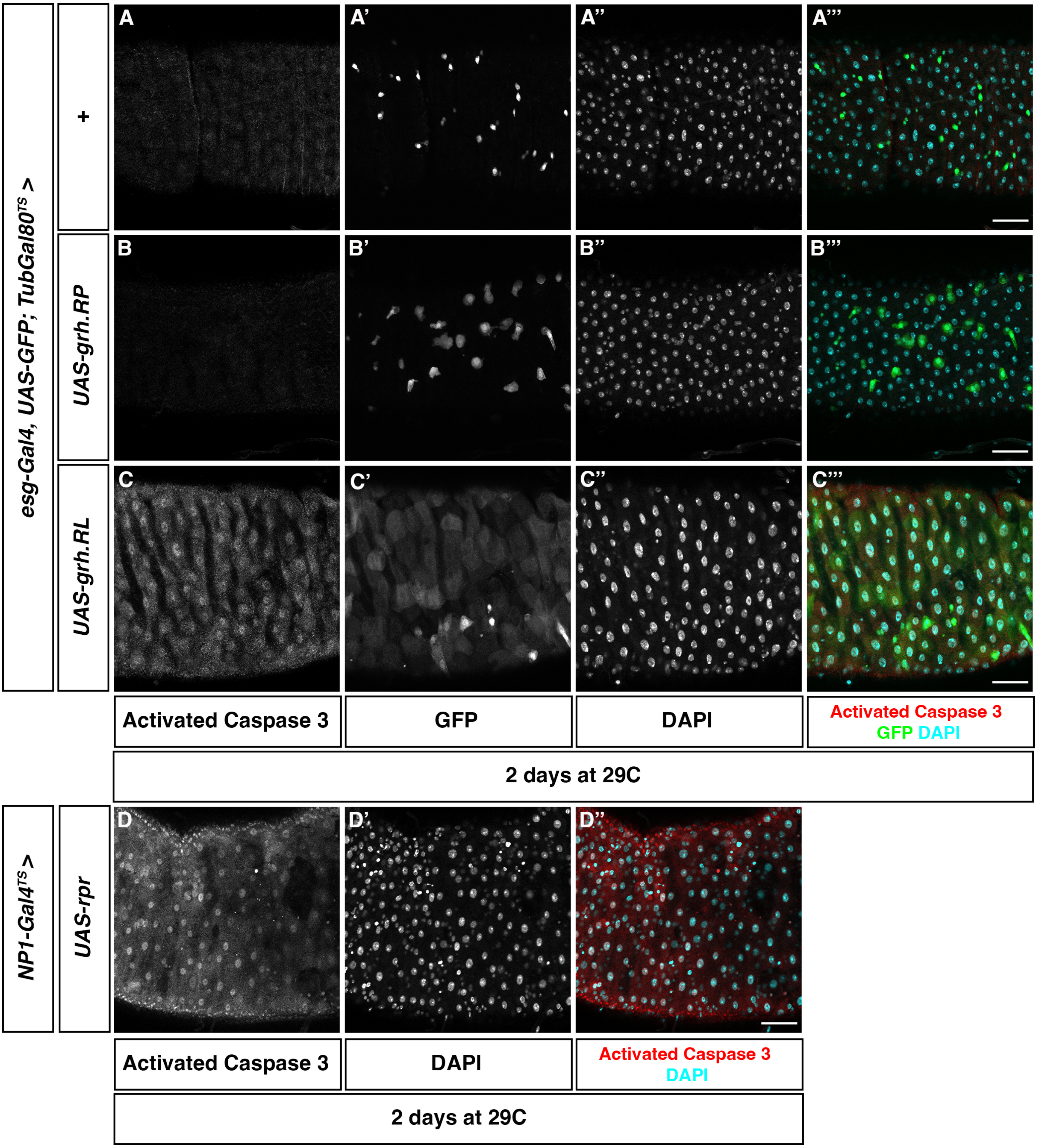

Supplementary figure 5

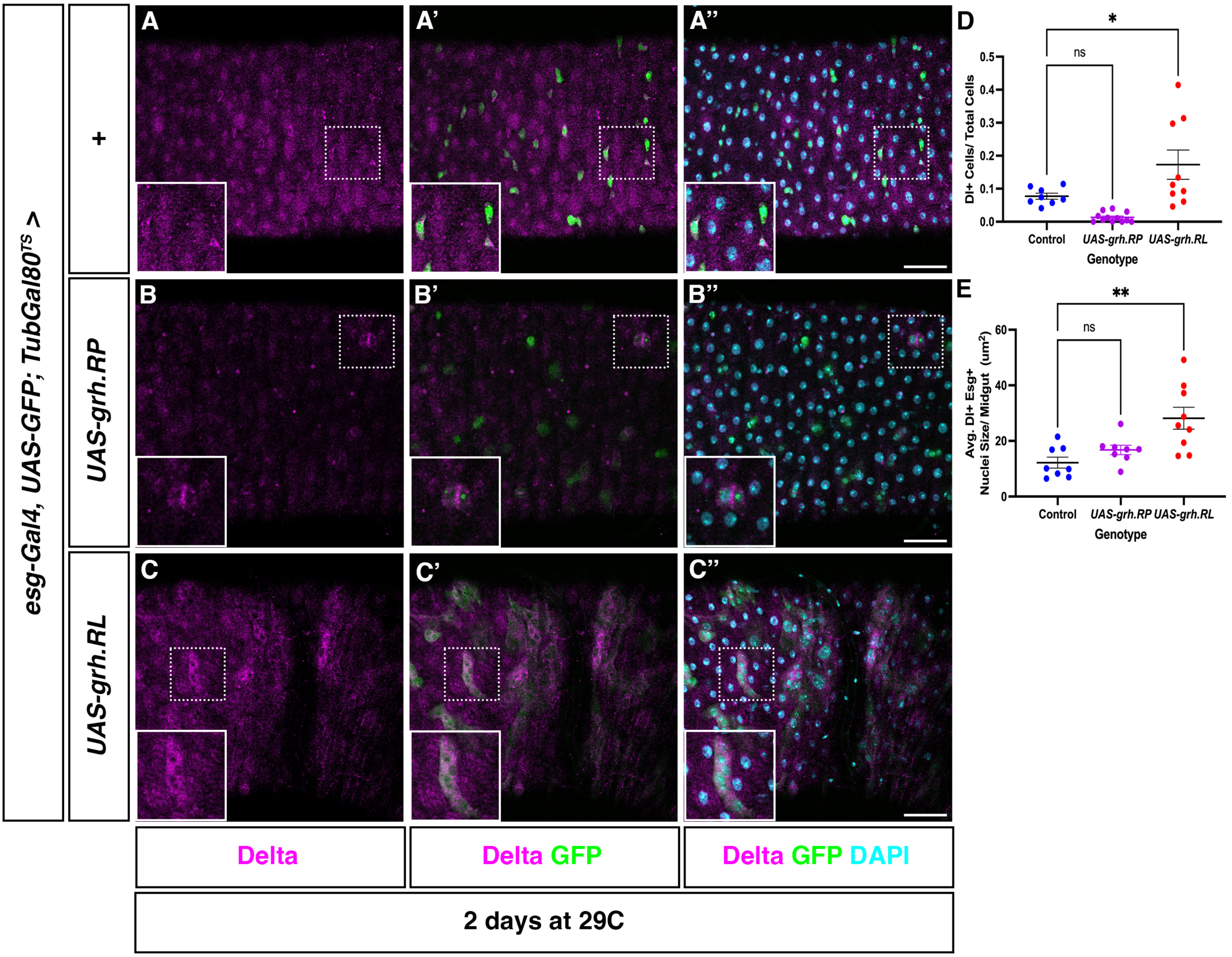

Supplementary figure 6

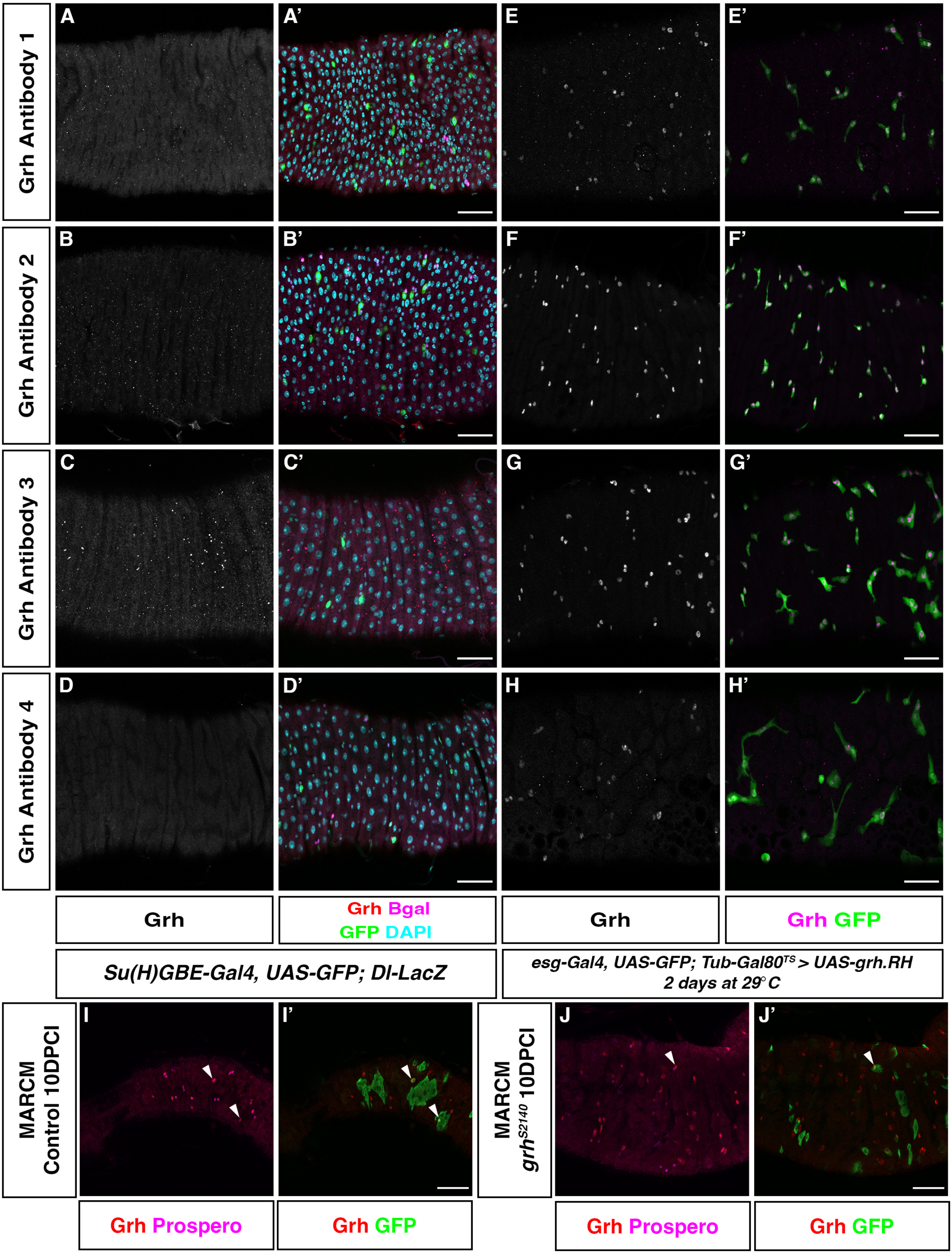

Supplementary figure 7

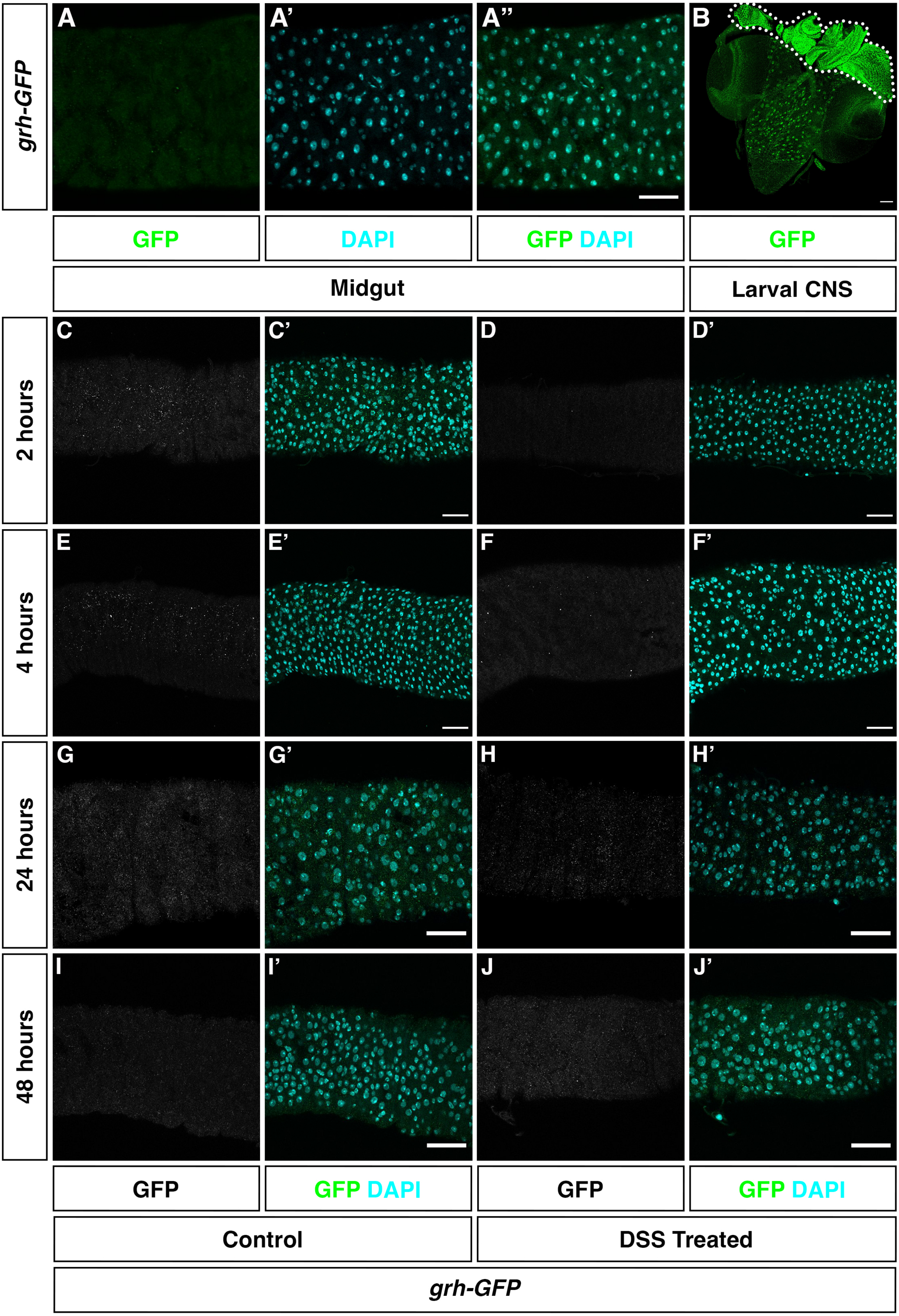

Supplementary figure 8

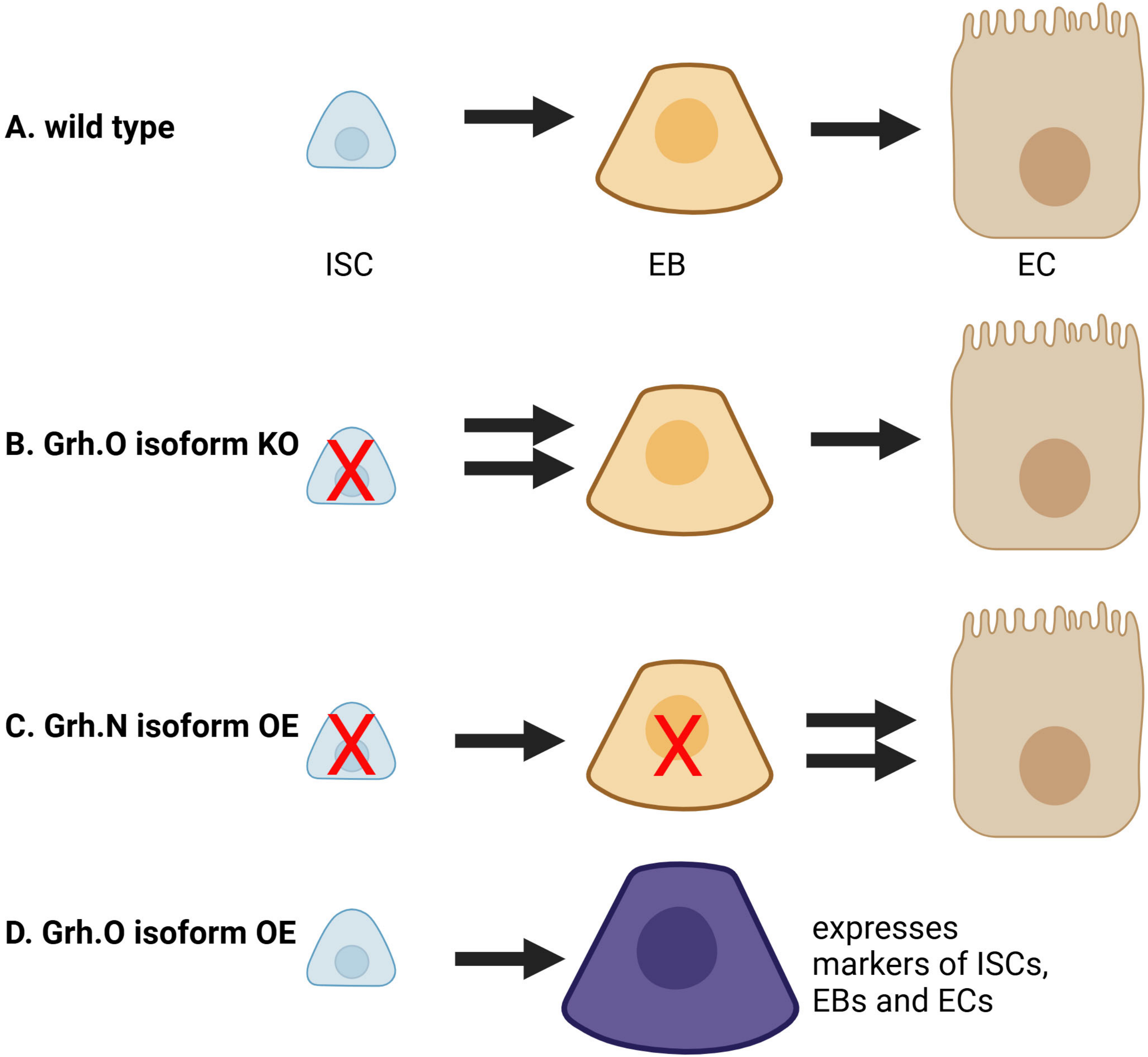

Supplementary Figure 9
